# Journal of Global Health’s Guidelines for Reporting Analyses of Big Data Repositories Open to the Public (GRABDROP): preventing ‘paper mills’, duplicate publications, misuse of statistical inference, and inappropriate use of artificial intelligence

**DOI:** 10.7189/jogh.15.01004

**Published:** 2025-07-01

**Authors:** Igor Rudan, Peige Song, Davies Adeloye, Harry Campbell

**Affiliations:** 1 Centre for Global Health, Usher Institute, The University of Edinburgh, Edinburgh, UK; 2 Nuffield Department of Primary Care Health Sciences and Green Templeton College, Oxford University, Oxford, UK; 3 School of Public Health, Zhejiang University, Hangzhou, China; 4 Centre for Public Health, School of Health and Life Sciences, Teesside University, Middlesborough, UK

## Abstract

In recent years, global accessibility to large ‘big data’ repositories that enable ‘open research’ – such as the UK Biobank, National Health and Nutrition Examination Survey (NHANES), and Global Burden of Disease (GBD) datasets – has created unprecedented opportunities for researchers worldwide to conduct secondary data analyses. This development is particularly beneficial for early-career researchers in low- and middle-income countries (LMICs), as it lets them access large and otherwise costly datasets without the need for local infrastructure, potentially curbing brain drain. However, through our work at the Journal of Global Health (JoGH), we have identified emerging concerns that must be addressed to help preserve the integrity and scientific value of this otherwise positive trend. These include: the risk of ‘paper mills’ mass-producing superficial papers with questionable authorship practices; duplicate publications produced through republishing already available results or by multiple groups testing the same hypothesis using identical datasets and methods without awareness of each other’s work; proliferation of false-positive findings due to inadequate adjustment for multiple testing in large datasets; and the inappropriate or undisclosed use of artificial intelligence (AI) tools in generating manuscripts. To counter these issues while continuing to support legitimate and innovative secondary data analyses, JoGH is introducing guidelines for authors submitting such work for consideration and peer review. These guidelines require authors to declare transparently: their previous published work based on similar datasets or hypotheses; the originality of their research question and design in the context of other similar research; their awareness of related published studies using the same dataset; how they addressed multiple testing statistically; and the role of AI, if any, in manuscript preparation or data analysis. A new, mandatory section in such submitted manuscripts – ‘Adherence to JoGH’s Guidelines for Reporting Analyses of Big Data Repositories Open to the Public (GRABDROP)’ – will summarise these declarations, with full details provided in a supplemental file. This proactive editorial policy aims to safeguard scientific quality while empowering global researchers. By improving transparency and accountability, JoGH seeks to ensure that the benefits of open big data are not undermined by unethical or careless practices. We suggest that other publishers engage in an open discussion on how to address these challenges and consider adopting JoGH’s GRABDROP guidelines or similar measures to maintain trust in scientific outputs derived from secondary analyses. Through these steps, JoGH remains committed to fostering reproducible and equitable global health research.

In recent years, several massive repositories of ‘big data’ have become available and open for further analyses to researchers globally, allowing them to typically obtain approval for secondary analyses wherever they could generate a valid hypothesis and explain how they plan to test it. Consequently, we noticed an increase in the number of papers submitted to the Journal of Global Health (JoGH) based on secondary analyses of such datasets, with the most common being the UK Biobank [1], the National Health and Nutrition Examination Survey (NHANES) [2], and the Global Burden of Disease (GBD) [3], alongside other international and national datasets which we encounter less frequently.

In principle, this should be a welcome practice that can significantly accelerate scientific progress. Opening such big data repositories to researchers globally maximises the potential for the harnessing of existing data to generate new knowledge. Another attractive element of this approach is that, across many low- and middle-income countries (LMICs), early and mid-career researchers now have an unprecedented opportunity to benefit from these data. They are the first generation of researchers from LMICs who can work freely on openly available datasets that were very expensive to generate, enabling them to conduct meaningful, independent research from within their own countries, where such datasets would typically be unfeasible to generate and maintain. This might also indirectly help prevent ‘brain drain’ and keep promising researchers in their own countries, thus building the capacity of their academic institutions. Publicly available datasets allow young researchers to apply their knowledge, test their hypotheses, and practice their analytical skills using real-world data (albeit with data from populations or settings that are not representative of LMICs), often without the financial and logistical burdens associated with fieldwork. This can enhance research productivity and build essential expertise in big data analysis – a skill increasingly critical across global health disciplines. At JoGH, we view this shift as a positive progress for biomedical and public health research.

In theory, this should really be a win-win scenario for all stakeholders. Funders of those datasets will see many more research outputs produced in a shorter time frame, and in some cases may even recover part of their investment through user fees. Scientists in high-income countries (HICs) who had originally developed those datasets will achieve broader impact throughout the world, while interested researchers everywhere, but particularly in LMICs, will get an unprecedented opportunity to consistently contribute to cutting-edge scientific discovery. This is especially true for early-career researchers who can be actively engaged and trained in such analyses, building skills relevant for future careers in academia or industry. More broadly, the global public stands to benefit as well, as this increased research activity helps fill critical evidence gaps in health, ultimately informing policy and practice across diverse settings, especially those lacking the capacity or funds to generate population-level datasets themselves.

## FIVE EMERGING CONCERNS

However, in practice, some unexpected issues began to arise. A recent review of 341 studies of the NHANES dataset raised concerns that many adopted single-factor analytical approaches for diseases where multifactorial analyses were warranted, failed to account for false discovery rates, or did not elaborate on their use of subsets of data [4]. Others have warned of the proliferation of low-quality papers that used two-sample Mendelian randomisation to analyse data from the UK Biobank, which is not strictly open to the general public for direct access, but rather all *bona fide* researchers who are undertaking health-related research in the public interest can apply to access the data [1]. The studies that are being scrutinised utilise genetic variants associated with an exposure of interest as proxies for the exposure and link it to a specific outcome, without explaining their reasoning or the underlying biological mechanisms or setting their findings properly in the context of other causal evidence [5,6]. Another question is to which extent can republished extracts from large supplementary documents and datasets that underpin GBD studies be viewed as original contributions, and to which extent they duplicate what has essentially been published in another form already. These phenomena raise concerns which we outline below – while we noticed these issues in our work at JoGH, they also reflect broader challenges faced by academic publishing and science in general.

### Paper mills with questionable authorships

The first concern is that some of these papers could originate from so-called ‘paper mills’ – initiatives that produce either fake or poor-quality papers, offering authorships to researchers who seek it for academic advancement, and sometimes going as far as offering peer-review or even direct publication in cooperation with some participating journals and/or publishers [7,8]. ‘Big data’ repositories are especially susceptible to these malpractices: paper mills, aided by artificial intelligence (AI), can basically reanalyse these datasets rapidly and produce low-quality papers for further misuse [4]. Aside from flooding science with irrelevant or misleading research, this leads to editors and reviewers being overwhelmed and struggling with recognising such papers. The problem has become so widespread that publishers, journals, academic organisations, and individuals joined into United2Act – a group dedicated to fighting this issue by systematically researching paper mills and developing possible countermeasures for all stakeholders [8]. To date, paper mills have been identified to arise from both large and small scientific communities, but mainly in countries where both regulation and implementation of ethical research practices may be lacking [9].

What drives researchers to take part in paper mills? One reason is the ‘publish-or-perish’ culture, which often predicates their academic advancement on quantity, rather than quality. Clinicians are often expected to publish professional and scientific papers to advance their careers, despite not having the time, interest, or data to do so. It is, therefore, not surprising that we see paper mills originating where researchers and clinicians are underfunded and overburdened, making it particularly challenging for them to conduct research of their own, whilst requiring many publications to advance professionally in their institutions [10–12].

At JOGH, we stand against such practices and are vigilant for papers that might be indicative of paper mills. We will investigate, in accordance with the practices suggested by organisations such as the Committee on Publication Ethics (COPE), any concerns raised about papers submitted or published in JoGH. Yet, based on our experiences with many authors from LMICs, we also suggest that, when concerns do arise, editorial decisions should not be influenced by stereotypes or assumptions that could lead to unfair scrutiny or rejection of submissions from institutions originating from such contexts. Such biases would penalise researchers who are conducting legitimate, high-quality secondary analyses, particularly those from those underfunded, underrepresented settings.

### Duplicate publications arising from ‘reorganising’ published information

Another concern is that some of those papers may essentially represent duplicate publications, but in two entirely different ways. The first scenario, addressed in this sub-section, is apparent and could be prevented; while the second one is situational and more challenging to address (see the next sub-section). In the first scenario, which is most applicable to analyses of the large datasets that underlie GBD estimation, authors extract the data or results on a specific disease or risk factor from those datasets in public domain. They typically obtain the data from the GBD website, rather than from the supplementary information in the large GBD publications, although the latter can also happen. Then, they reorganise the extracted information to present it as a new, stand-alone paper related to a specific topic. At JoGH, we consider GBD publications to be comprehensive, in the sense that they include all the published text, the full supplementary material, and the underlying datasets. Therefore, when the authors simply repackage previously published information without offering new insights or analytical novelty or addressing additional research questions, JoGH considers this a duplicate publication which should not be considered. This means that we are only open to reviewing submissions that use GBD data in a legitimate way, to test novel hypotheses and/or employ improved or new methods [3].

### Duplicate publications arising from parallel work of multiple groups

The other scenario related to duplication, regardless of which dataset is used, is one where two or more groups of authors are testing the same hypothesis using the same dataset and methods. We want to ensure, when reviewing an article, that the authors are aware of all previous or parallel attempts by other groups to address the same research question using the same dataset. This is why we will ask them to declare all such studies that they are aware of and to cite them in the introduction to their paper if they have already been published.

### Misuse of statistical inference: false-positive results from multiple testing

The next concern is that, in massive ‘big data’ repositories, so many possible hypotheses can be tested using the data that there could be many false positive associations around which a paper could be constructed. Reporting such associations is of very low value, unless a rigorous statistical method that accounts for multiple testing, such as Bonferroni correction, has been applied [13,14]. One type of such papers that we see frequently submitted are those based on Mendelian randomisation approach which, in the way they are being conducted, may be biased towards positive findings [6].

### Inappropriate use of AI

The fifth concern is that this kind of malpractice can be facilitated by AI and large language models. With ‘big data’ repositories opening to AI- or machine learning-driven analyses [15,16], researchers have raised concerns about their misuse and a potential overflow of redundant or low-quality papers based on these large datasets [4].

In 2023, JoGH issued a position statement on the use of AI in research [17], with one key aspect being the authors responsibility ‘for the work performed by a chatbot in their paper (including the accuracy of what is presented, and the absence of plagiarism) and for appropriate attribution of all sources (including for material produced by the chatbot)’ [17].

This means that an increasing portion of the text in these papers, and possibly even the contents of tables and figures, will be generated by the currently available AI chatbots. As the original datasets are enormous in terms of their size and the steps that the AI then takes may not be easy to retrace, the editors and reviewers may not be able to spot any errors – not even the important ones that may change the message of the paper entirely. There is no obvious way for editors or reviewers to know whether some mistakes may exist in such complex analyses of such large datasets. We can only trust the authors that they did all the work and that others can replicate it. Yet if their main motivation was to simply generate many papers to gain some advantage with no regard to scientific accuracy, then many false papers may end up getting published. Concerns have been raised that this is already detectable [12,15].

## EDITORIAL POSITION AND ACTIONS OF JOGH ON THESE FIVE EMERGING CONCERNS

In this editorial, we seek to explain our position towards these new challenges with secondary data analyses and describe how we propose to tackle these issues when handling manuscripts submitted to JoGH. We have formulated this as a set of recommendations which may be useful for other journals to consider. We will assume that all our authors who submit manuscripts to JOGH do so free from any of these questionable practices unless there is evidence to the contrary. However, we think that there is enough evidence in the scientific literature of these practices becoming more widespread that it would be prudent for us to take actions to protect the Journal of Global Health and the scientific literature against them. We aim to do this with an approach that is not overly burdensome for authors when submitting manuscripts so as not to discourage the great majority of submissions reporting legitimate research. A first step in this direction was introduced in late 2023, when we began asking all our authors within the submission process to declare that their paper is not part of a ‘paper mill’. However, reflecting on our experience with this evolving issue, we now believe we can improve our response to these challenges. With this in mind, we present our Guidelines for Reporting Analyses of Big Data Repositories Open to the Public (GRABDROP). We publish them here, for all our prospective authors of secondary analyses of big data to use.

We will ask our authors to declare within their submission:

- other similar secondary analyses which they have co-authored and where (to address concerns about the issue of ‘paper mills’);
- what makes their study truly original (to prevent duplicate publication);
- all other secondary analyses similar to theirs that have been published by other groups and that they are aware of, and whether they have been cited properly;
- how are they addressing the issue of multiple testing through appropriately rigorous statistical thresholds of significance;
- to what extent have AI chatbots been used in producing their papers, and to which parts of the text they contributed.

We consider these to be necessary steps for a publisher to take under the current circumstances. By their nature, they are primarily meant to protect the authors who may be unaware of risks involved from these inappropriate or questionable practices, and also the scientific journals. We will require all authors of these papers to add a short section between their ‘Introduction’ and ‘Methods’ called ‘Adherence to JoGH’s Guidelines for Reporting Analyses of Big Data Repositories Open to the Public (GRABDROP)’, and then provide a link to a table in their Online Supplementary Document in which they address all these concerns. We provide a template for this table below (**Table 1**). We will also make our reviewers aware of these guidelines, and work on improving editors’ and reviewers’ skills to detect related issues that our editorial review may have missed. Our editorial team is keeping in touch with trends and tools in this context. We will review our experience with this approach (and that of other journals) in the coming year and will update it as required.

**Table 1:** Outline of JoGH’s Guidelines for Reporting Analyses of Big Data Repositories Open to the Public (GRABDROP) items

JoGH guideline item	Purpose
1. Please list all papers published by each co-author in previous three years that were based on secondary analysis of a big data repository	Preventing publications from possible ‘paper mills’ and authorships granted without genuine contribution
2. Please explain the key elements of your study design and the use of the available datasets that make your study an original scientific contribution	Preventing duplicate publication where published information is reorganised and republished
3. Please list all publications that addressed similar research questions in the same dataset and indicate where you cited them in your paper	Preventing duplicate publication where other groups have already conducted similar research in the same dataset
4. Please explain how you addressed multiple testing through an appropriately rigorous statistical threshold and indicate this in the methods section	Preventing publication of false positive associations which are of low value unless a rigorous statistical method accounted for multiple testing
5. Please declare to what extent have AI chatbots been used in developing your paper and to which parts of the paper did they contribute	Preventing the improper use of AI chatbots in developing the key elements of the paper and ensuring reproducibility and transparency

AI – artificial intelligence, JoGH – Journal of Global Health

## CONCLUSIONS

We believe that these key concerns can all be addressed effectively through the use of JoGH's guidelines under the assumption that authors report transparently and honestly. These statements would reassure us that they are not adopting any questionable practices. Then, we would submit their papers for peer review like any other and, given the reviewers’ positive assessment, would have no additional reservations to publishing their work.

Another challenge that could then result from increasingly frequent secondary analyses of big data repositories, which is not going to be easy to address, is that researchers in LMICs may increasingly rely on large datasets from HICs to build their careers. While this can provide valuable research opportunities and allow them to develop analytic skills, it may also diminish the incentive to develop and invest in local data collection essential for addressing local health priorities. This is particularly problematic for early- and mid-career researchers under pressure to publish, who may view secondary analyses of ‘big data’ as a faster and easier route to academic advancement. Over time, this reliance could hinder capacity building in LMICs and shift research focus away from local needs.

Moreover, HIC datasets may not be representative of LMIC populations and settings and thus findings derived from these data may not be directly applicable to LMIC populations, potentially undermining the policy relevance of the evidence produced. There is also a risk of misinterpretation whenever researchers unfamiliar with the context of the dataset, such as the health system, cultural factors, or socioeconomic conditions, draw conclusions or make policy suggestions. To advance global health research further, it is essential that LMIC-based researchers are empowered to generate, interpret, and share their own ‘big data’. These researchers possess critical contextual knowledge and are best positioned to produce insights and recommendations that are both accurate and locally relevant. We therefore support greater investment in building large, open-access data infrastructures in LMICs, led by local experts and accessible to the global research community.
